# Sexual precocity with pituitary macroadenoma and bilateral multicystic ovaries - a case report

**DOI:** 10.1186/1687-9856-2013-S1-P200

**Published:** 2013-10-03

**Authors:** S Ramkumar, Parjeet Kaur, Ansu Joshi, AC Ammini,

**Affiliations:** 1 Dept of Endocrinology and Metabolism, AIIMS, New Delhi, India

## Aim

To report a 6 years old girl who presented with sexual precocity, multicystic ovaries and pituitary macroadenoma due to primary hypothyroidism.

## Methods

We describe the clinical presentation, imaging findings, hormonal work-up and follow up of a child with sexual precocity, multicystic ovaries and pituitary macroadenoma due to primary hypothyroidism

## Results

6 year old girl presented with vaginal bleeding for last 1 ½ years. The initial episode lasted 3 days. The vaginal bleeding then continued every month lasting 3-4 days for next 9months. She also had inter-menstrual bleeding for last 6 months. Parents also noticed breast development (initially left followed by right a month later) for the past 6 months. There was no galactorrhoea, pubic or axillary hair development. There was no height gain during the last 2 years, but weight increased from 14kg to 19kg. Her appetite was normal. Parents also noticed a generalized edema of face, abdomen and legs. There was no history of constipation, cold intolerance or goiter. Her past history was unremarkable. She goes to Anganwadi School but has decreased mentation compared to younger sister. On examination, she was 95.5 cm tall (<3^rd^ percentile), weight was 19 kg (25^th^ percentile ). Her skin was rough and dry, heart rate was 80/min and BP 100/80mm of Hg. There was no goiter or lingual thyroid. Tanners staging was B3P1. Galactorrhoea present on gentle manipulation. There was no axilliary hair, genital hyperpigmentation or clittoromegaly. Her cardiac and respiratory examinations were normal. On abdominal examination, a soft cystic fluctuant mass palpable in hypogastrium, dull on percussion, well defined on all sides except inferiorly where lower margin was not palpable. Neurological examination showed dull lethargic child with excessive somnolence. The tendon reflexes are normal. Her bone age was delayed 3.2 years. Investigations: Hemogram, renal and liver functions are normal. Her hormonal profile: Total T4 0.549µg/dl (5.1-14.1), TSH > 100 µIU/ml (0.27-4.2), TPO antibody – 74.01 pg/ml (<34), LH <0.10(2.4-12.6), FSH 7.78mIU/ml(3.5-12.5), prolactin 337.7ng/ml(6-29.9), Estradiol 2113pg/ml(<20), Cortisol 15.95µg/dl(6.2-19.4), testosterone 0.029ng/ml(0.084 – 0.481), DHEAS 39.37µg/dl(2.8-85.2), ACTH 11.71 pg/ml (7.2 – 63.3). USG neck revealed presence of thyroid in normal location. USG and CT pelvis showed bilateral multiple cystic ovaries and bulky uterus. MRI sella reported as pituitary macroadenoma. Child was started on tab.thyroxine 50 mcg/day. Child lost 4 kg and gained 1.5cm in 2 months and became active. Repeat hormonal work up showed: Total T4 10.59 µg/dl, TSH 3.18µIU/ml and prolactin 71 ng/ml. she was diagnosed as hashimoto thyroiditis/hypothyroidism with pituitary macroadenoma and sexual precocity.

## Results

Short stature in sexual precocity - think of primary hypothyroidism. Multi cystic ovaries and pituitary enlargement although rare, may be seen in children with longstanding primary hypothyroidism. It is important to be aware of this to avoid pituitary surgery which may have disastrous results in these patients.

